# Chronic liver disease-related osteoporosis: From mechanisms to precision intervention

**DOI:** 10.1016/j.jhepr.2026.102029

**Published:** 2026-09-15

**Authors:** Wenjing Wang, Guangyu Liu, Burhan Yokus, Taiqiang Yan, Haolin Sun, Pal Pacher, Yan Wang

**Affiliations:** 1 Department of Infectious Diseases, Peking University First Hospital, Beijing, 100034, China; 2 Laboratory of Cardiovascular Physiology and Tissue Injury, National Institute on Alcohol Abuse and Alcoholism, National Institutes of Health, Bethesda, MD, 20892, USA; 3 Department of Orthopedics, Peking University First Hospital, Beijing, 100034, China; 4 Orthopaedic Department, Taiyuan Central Hospital, Taiyuan, Shanxi Province, China

**Keywords:** Hepatic osteodystrophy, RANKL–RANK–OPG signaling, Osteoclastogenesis, Trabecular bone score, Bone turnover markers, Bile acid signaling, IGF-1/IGFBP-1 signaling

## Abstract

Patients with chronic liver disease (CLD) face a markedly increased risk of osteoporosis and fragility fractures, but this systemic complication remains insufficiently recognised, while conventional assessment based mainly on bone mineral density may underestimate clinically relevant alterations in bone quality and microarchitecture. By synthesising epidemiological data and both shared and aetiology-specific mechanisms across chronic hepatitis B, metabolic dysfunction-associated steatotic liver disease, alcohol-associated liver disease, and autoimmune hepatitis, this review offers an integrated framework for hepatologists, endocrinologists, and translational researchers. The evidence highlights a multifactorial pathogenesis involving chronic inflammation, endocrine and metabolic dysregulation, and disturbances in the liver-bone and liver-gut-bone axes, which converge on impaired osteoblast–osteoclast homeostasis and progressive bone loss. The evidence reviewed underscores the value of risk-stratified screening, dynamic assessment of bone quality and biomarkers, and individualised preventive and therapeutic strategies to optimise skeletal health in patients with CLD. Emerging approaches, including bone-targeted drug delivery and therapeutic modulation of cross-organ metabolic signalling, may further expand opportunities for precision management. However, because a substantial proportion of the mechanistic evidence remains preclinical, these translational implications should be interpreted cautiously and require confirmation in well-designed prospective multicentre studies prior to widespread implementation.

## Graphical abstract

**Figure:**
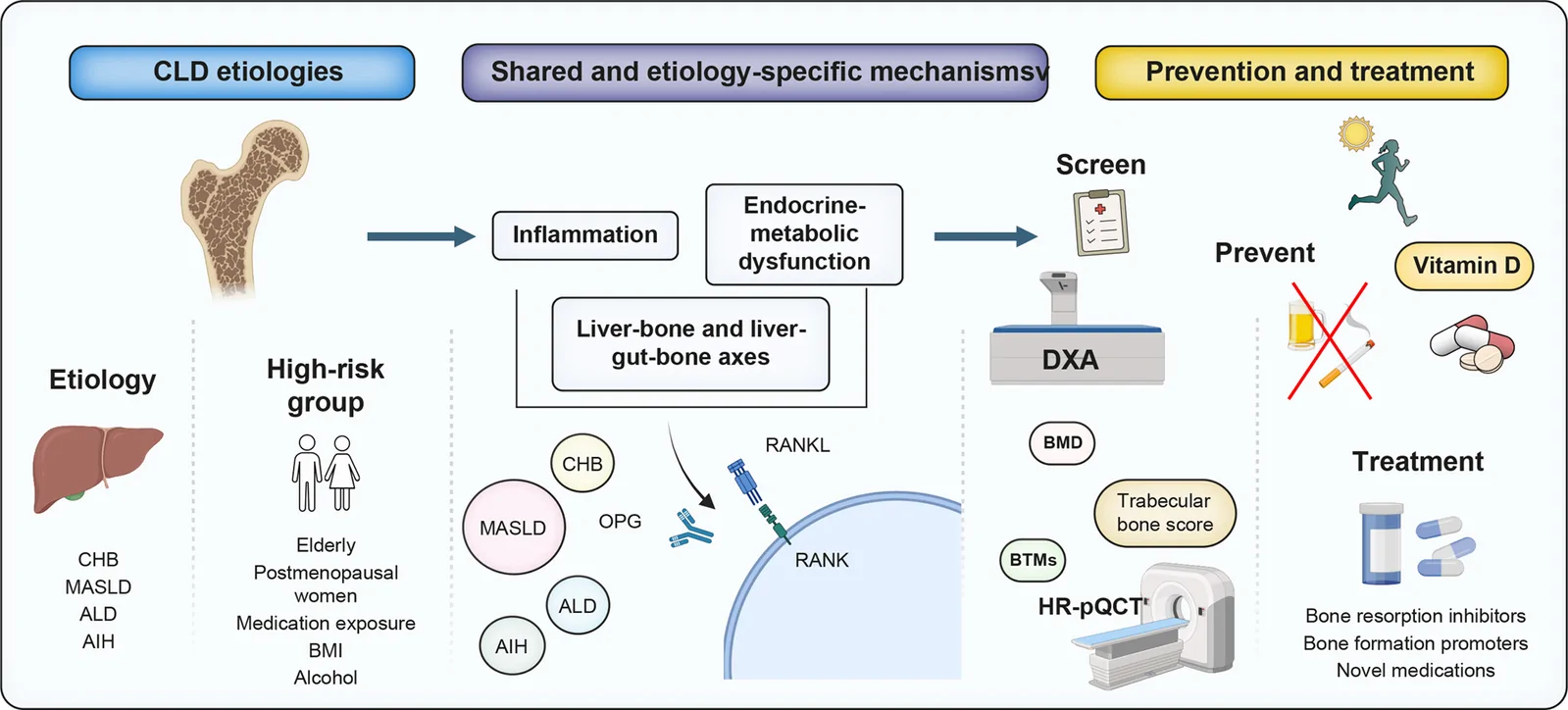

**Keypoints**

• Chronic liver disease-related osteoporosis is an underrecognised extrahepatic complication that increases fragility fracture risk and worsens long-term outcomes.
• Osteoporosis occurs across major chronic liver disease aetiologies, including chronic hepatitis B, metabolic dysfunction-associated steatotic liver disease, alcohol-associated liver disease, and autoimmune hepatitis.
• Disease severity, hepatic fibrosis, cirrhosis, aging, postmenopausal status, low bone mineral density, alcohol exposure, glucocorticoids, and selected antiviral therapies increase skeletal risk.
• Shared mechanisms include osteoclast-osteoblast imbalance, inflammation, oxidative stress, endocrine-metabolic dysregulation, and disruption of the liver-gut-bone axis.
• Aetiology-specific mechanisms may further shape skeletal risk, including viral immune activation, lipotoxicity, alcohol-induced osteoblast dysfunction, and autoimmune or glucocorticoid-related bone loss.
• Bone mineral density alone may underestimate fracture risk because it does not fully capture bone quality, microarchitecture, or dynamic remodelling abnormalities.
• Risk-stratified assessment should integrate clinical risk factors, DXA, trabecular bone score, HR-pQCT where available, bone turnover markers, and vitamin D status.
• Management should combine liver disease control, lifestyle optimisation, fall prevention, calcium and vitamin D correction, and individualised anti-osteoporotic therapy.

## Introduction

Chronic liver disease (CLD) represents a major global health challenge. Advances in vaccination, antiviral therapy, and clinical diagnostics have improved patient survival, shifting disease management from isolated hepatic lesion control toward long-term care focused on multisystem complications. Among these, liver disease-related osteoporosis substantially increases the risk of fragility fractures, severely impacting patients' quality of life and carrying significant clinical implications. Epidemiological data suggest that osteoporosis is more prevalent in patients with CLD than in healthy populations, with reported prevalence estimates of approximately 20%-50% in CLD cohorts^1^^,^^2^ and an overall risk that is approximately 1.93-fold higher than that of healthy controls.^3^ Importantly, fractures in patients with advanced liver disease are not merely skeletal events but are closely linked to adverse clinical outcomes. For example, the 1-year all-cause mortality after hip fracture has been reported to reach 26.4% in patients with cirrhosis,^4^ and to exceed 50% in those with decompensated disease.^5^ These observations highlight the critical importance of bone health assessment and intervention as part of comprehensive CLD care.

The pathogenesis of bone loss in CLD is complex and multifactorial. Distinct aetiologies, including chronic hepatitis B (CHB),^6^ metabolic dysfunction-associated steatotic liver disease (MASLD),^7^ alcohol-associated liver disease (ALD),^8^ and autoimmune hepatitis (AIH), may promote skeletal fragility through both shared and aetiology-specific pathways. These mechanisms involve chronic inflammation, immune dysregulation, endocrine and metabolic disturbances, and disruption of signalling networks within the liver-gut-bone axis. Furthermore, commonly used clinical interventions, such as glucocorticoids and nucleoside analogues including tenofovir disoproxil fumarate (TDF) and adefovir dipivoxil, further exacerbate bone loss.

Although previous studies have linked liver diseases with osteoporosis, the shared and aetiology-specific mechanisms have not been sufficiently integrated. This limitation hinders the development of precise risk stratification and targeted prevention strategies in clinical practice. This review aims to synthesise recent evidence on liver disease–related osteoporosis with a focus on four major CLD aetiologies: CHB, MASLD, ALD, and AIH. Within a framework encompassing inflammatory responses, endocrine–metabolic dysregulation, and perturbations of the liver–gut–bone axis, we summarise current epidemiological evidence and mechanistic insights. Furthermore, we discuss risk-stratified screening approaches and propose aetiology-oriented prevention and treatment strategies, while highlighting emerging therapeutic directions and future research priorities. Collectively, this review aims to provide a clinically relevant framework for improving bone health management in patients with CLD.

## Epidemiology of liver disease-related osteoporosis

### Prevalence and incidence

CLD is strongly associated with an increased risk of low bone mineral density (BMD) and fragility fractures. In particular, patients with cirrhosis have been reported to have approximately 1.93-fold higher odds of osteoporosis than non-cirrhotic controls.^3^ Although low BMD is observed across multiple CLD aetiologies, the magnitude and pattern of skeletal involvement vary according to both disease severity and aetiology. HBV infection has been identified as an independent risk factor for osteoporosis.^9^ A retrospective analysis of 180,730 individuals revealed that patients with CHB had a 1.13-fold higher risk of osteoporosis compared to healthy controls.^10^ Mendelian randomisation studies further support a causal relationship between HBV infection and osteoporosis from a genetic perspective.^9^ Previous studies have demonstrated that HBV-infected patients exhibit significantly lower BMD in the lumbar spine, total hip, and femoral neck than healthy individuals,^11^ and HBsAg positivity has also been associated with lower BMD.^12^ In HBV-infected patients, fractures commonly occur in the spine and hip, consistent with the anatomical distribution characteristics of typical osteoporotic fractures.^13^

In Asian populations, MASLD has been associated with a 35% increased risk of osteoporosis and a 31% higher risk of osteoporotic fractures.^14^ Moreover, compared to patients with MASLD, patients with ALD exhibited more significant damage to bone microstructure.^15^ Patients with ALD showed reductions in average calcium content, microhardness, and structural damage in the bone matrix in both the lumbar spine and femoral neck,^16^ with the most pronounced damage observed in the trabecular bone of the lumbar spine.^17^ Patients with AIH also experienced significantly elevated fracture risks, with an incidence rate of approximately 17.45/1,000 person-years, 1.24-fold higher than that in healthy individuals.^18^ Imaging studies have shown significant decreases in the cortical thickness of the distal radius and tibia in patients with AIH.^19^ Collectively, while the specific patterns of skeletal degradation vary by underlying hepatic aetiology, compromised bone health remains a universal complication of CLD. This pervasive epidemiological burden underscores the necessity of identifying distinct high-risk cohorts and the specific disease-related clinical factors that drive accelerated bone loss.

### High-risk groups and risk factors for osteoporosis

The development of liver disease-associated osteoporosis is driven by both intrahepatic bone metabolic disorders and extrahepatic bone fragility (Fig. 1). The severity of structural and functional hepatic impairment serves as a core determinant of bone loss, encompassing not only histologic fibrosis progression,^18^^,^[20], [21], [22], [23], [24] but also non-invasive markers of liver injury and stiffness, such as the fibrosis-4 index and liver stiffness measurement. Compared with compensated cirrhosis, decompensated cirrhosis is typically associated with more rapid hepatic functional decline, a higher risk of falls, and more pronounced bone loss. Clinical evidence supports a dose-dependent relationship between fibrosis stage (F0–F4) and osteoporosis risk. Each 1-kPa increase in liver stiffness is associated with a 15% increase in the risk of osteoporosis.^25^ Notably, patients with MASLD and significant liver fibrosis assessed by transient elastography had 4.10-fold higher odds of osteoporosis than those without significant fibrosis.^26^ Beyond structural injury, worsening liver dysfunction can directly disrupt bone metabolism. As the Child-Pugh score progresses from A to C, bone loss gradually worsens.^27^

**Fig. 1:**
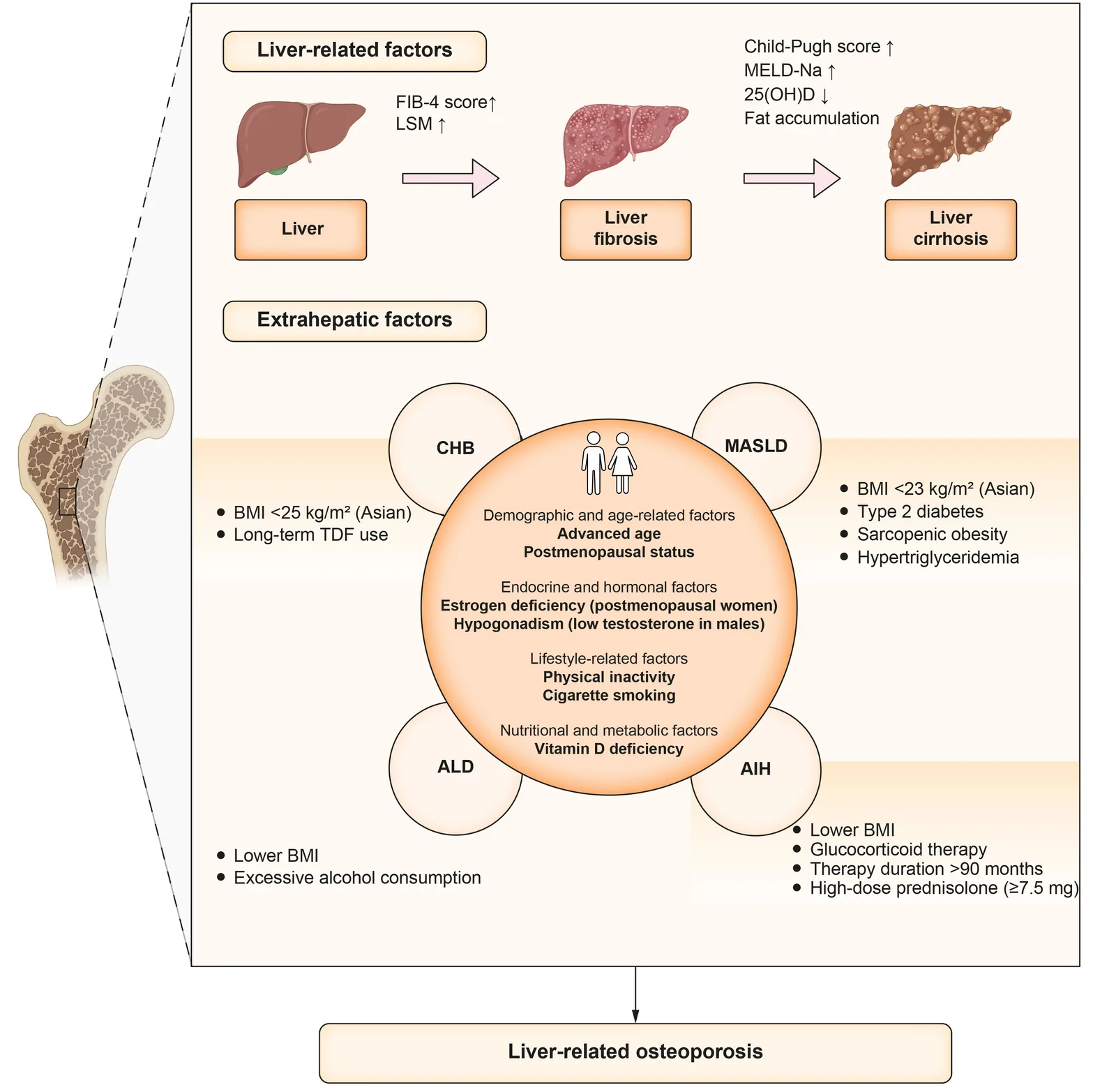
Risk factors of liver-related osteoporosis. This figure summarises the major risk factors contributing to the development of liver-related osteoporosis. The upper panel shows intrahepatic factors, indicating that as liver disease gradually progresses from early liver injury to liver fibrosis and cirrhosis, the risk of osteoporosis correspondingly increases. The lower panel presents extrahepatic factors, including both aetiology-specific risk factors and shared systemic factors (Central circle). 25(OH)D, 25-hydroxyvitamin D; AIH, autoimmune hepatitis; ALD, alcohol-related liver disease; BMI, body mass index; CHB, chronic hepatitis B; FIB-4, fibrosis-4; LSM, liver stiffness measurement; MASLD, metabolic dysfunction-associated steatotic liver disease; MELD-Na, model for end-stage liver disease-sodium; TDF, tenofovir disoproxil fumarate.

Extrahepatic factors, including biological sex,^11^^,^^28^^,^^29^ advanced age,^1^^,^^18^^,^^19^^,^^23^^,^^30^ low BMI,^1^^,^^31^ and prolonged exposure to medications such as certain antiviral drugs, proton pump inhibitors, and glucocorticoids,^1^^,^^2^^,^^19^^,^^23^^,^^32^ can further amplify osteoporosis risk to varying degrees. Aging-related systemic changes can interact with CLD. Aging is characterised by a state of chronic low-grade inflammation, which not only exacerbates the hepatic inflammatory milieu but also disrupts bone homeostasis through the accumulation of senescence-associated secretory phenotype factors, thereby promoting bone loss and skeletal fragility.^33^ Sex hormone deficiency further accelerates this process. In women, the gradual decline in oestrogen levels during perimenopause results in accelerated postmenopausal bone loss.^34^ In men with CLD, factors such as low testosterone levels and chronic alcohol consumption also significantly increase fracture risk.^35^ The effect of BMI on skeletal health is more complex. Compared to individuals with normal or low BMI, those with high BMI tend to have greater skeletal muscle mass and higher mechanical loading, which may confer partial protection against bone loss.^36^ However, high BMI is associated with vitamin D insufficiency^37^ and higher fall risk, which negatively impacts bone health.^31^ Notably, alcohol demonstrates direct, dose-dependent toxic effects on osteoblasts. Rather than conferring skeletal protection in patients with ALD, obesity exerts a synergistic deleterious effect with alcohol, thereby exacerbating bone destruction.^38^ This synergistic adverse effect underscores the unique clinical susceptibility of patients with metabolic dysfunction– and alcohol-associated steatotic liver disease (MetALD), in whom the combined burden of metabolic derangement and alcohol-induced hepatotoxicity may drive a markedly accelerated course of skeletal microarchitectural deterioration.^39^

## Mechanisms of liver disease-associated osteoporosis

Under normal physiological conditions, bone metabolism is maintained in a dynamic equilibrium between osteoblast-mediated bone formation and osteoclast-mediated bone resorption. CLD disrupts this homeostasis through multifactorial mechanisms, accelerating the shift toward bone loss and the progression of osteoporosis.

### Osteoclast-osteoblast remodelling imbalance

A hallmark of hepatic osteodystrophy is the disruption of the balance between osteoclast- and osteoblast-mediated bone remodelling. The receptor activator of NF-κB ligand (RANKL)-receptor activator of NF-κB (RANK)-osteoprotegerin (OPG) axis serves as the core pathway regulating osteoclastogenesis and activation, with its effects determined by the dynamic ratio of RANKL to OPG.^40^ Chronic inflammation and hepatic dysfunction can lead to a significant downregulation of OPG levels alongside an upregulation of RANKL expression, resulting in pathological osteoclast hyperactivity.^41^^,^^42^ This systemic dysregulation is further amplified in the inflammatory aging microenvironment, where the upregulation of p53 expression in hepatocytes suppresses the transcription of the miR-17 family, relieving its inhibitory control over RANKL and releasing excessive liver-derived RANKL into the circulatory system.^43^^,^^44^ Clinical studies have demonstrated that patients with CLD exhibit dysregulation of the RANKL/OPG system, which may contribute to impaired bone remodelling and reduced BMD.[45], [46], [47] Therefore, the serum RANKL/OPG ratio may represent a candidate biomarker for impaired bone remodelling and fracture susceptibility in CLD, although its independent predictive value requires prospective validation.

### Defects in the liver-bone and liver-gut-bone axes

The liver-bone axis provides a systemic framework for understanding bone loss in CLD by linking hepatic endocrine and metabolic disturbances to altered bone homeostasis.^2^

Among liver-derived endocrine mediators, the growth hormone (GH)–insulin-like growth factor-1 (IGF-1) axis is a critical anabolic signal. Hepatic injury reduces anabolic IGF-1 synthesis and simultaneously increases the secretion of insulin-like growth factor-binding protein 1 (IGFBP-1). Beyond modulating IGF-1 bioavailability, IGFBP-1 directly promotes osteoclastogenesis by binding integrin β1 on osteoclasts, enhancing RANKL-induced ERK phosphorylation and NFATc1 activation, and upregulating osteoclast-related genes.^48^ Impairment of this pathway is further compounded in elderly patients with CLD (aged ≥60 years) due to natural age-related declines in the GH–IGF-1 axis.^49^

In parallel, the role of the liver in regulating bone metabolism through lipid metabolic pathways should not be overlooked. Chronic liver injury upregulates the expression of protein phosphatase 2A, inhibits the production of lecithin-cholesterol acyltransferase, and obstructs the reverse cholesterol transport pathway.^50^ This metabolic disruption leads to the local accumulation of cholesterol and its oxidised products (such as 7-ketocholesterol and 27-hydroxycholesterol) within bone tissue, activating the reactive oxygen species (ROS)-transcription factor EB signalling pathway. This enhances osteoclast autophagy activity and bone resorption,^51^^,^^52^ while suppressing the expression of the osteoblast differentiation markers Runx2 (runt-related transcription factor 2) and alkaline phosphatase, thereby bidirectionally impairing bone formation. Advanced organoid co-culture models have revealed bidirectional pathways linking liver and bone aging.^53^^,^^54^ Aged liver organoids can induce senescence in bone organoids and accelerate bone loss through lipotoxic factors such as 27-hydroxycholesterol, while senescent bone organoids can in turn exacerbate liver dysfunction.^55^

It is worth emphasising that the liver-bone axis does not exist in isolation, and the liver-gut-bone axis also plays an important disease-specific role in CLD. Altered bile acid metabolism,^56^^,^^57^ intestinal barrier dysfunction,^58^ and gut microbiota dysbiosis^59^^,^^60^ synergistically drive systemic inflammatory responses and abnormal osteocyte remodelling. Chronic liver injury suppresses the farnesoid X receptor signalling pathway, disturbing enterohepatic circulation and reducing the production of secondary bile acids such as deoxycholic acid and lithocholic acid.^50^ Because secondary bile acids act as endogenous TGR5 ligands, their reduction may compromise osteoblast differentiation and weaken inhibition of osteoclastogenesis^61^.^62^ Consequently, the loss of these protective ligands, combined with cholestasis, impairs intestinal epithelial integrity by altering tight junction protein expression, promoting endotoxin translocation.^63^ This activates hepatic Kupffer cells and peripheral immune cells, amplifying systemic inflammatory mediators, including IL-6 and TNF-α.^64^ This gut-derived inflammatory amplification serves as an important intermediary through which liver dysfunction aggravates skeletal remodelling abnormalities (Fig. 2).

**Fig. 2:**
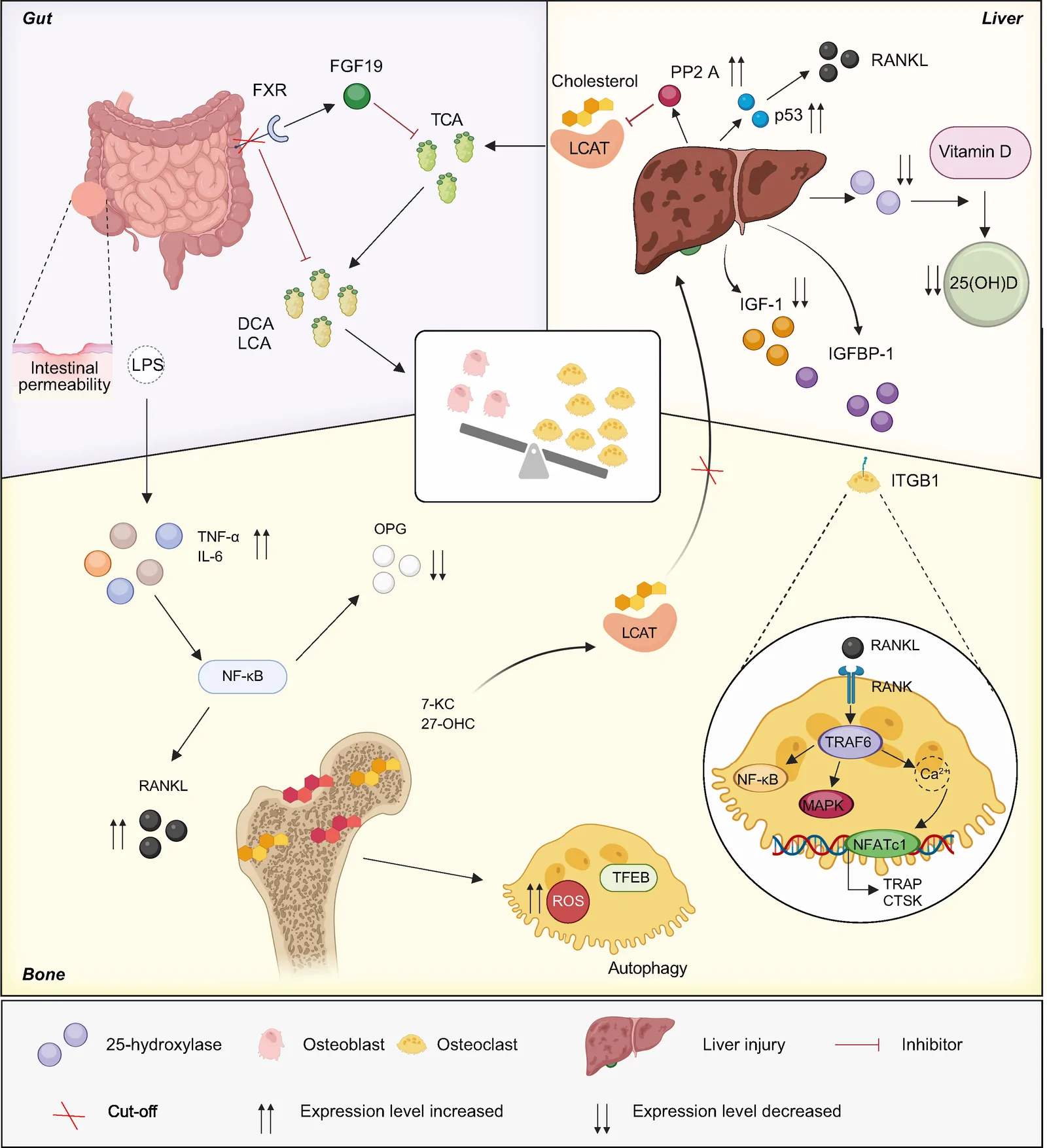
Liver-gut-bone axis. This figure summarises the main mechanisms by which liver-gut-bone axis dysfunction leads to bone loss in CLD. In the gut (upper left), impaired barrier integrity and increased permeability promote LPS translocation and systemic inflammation. Concurrently, altered bile acid signalling (FXR/FGF19) enhances the production of inflammatory cytokines and disrupts the RANKL/OPG balance, thereby promoting osteoclastogenesis. In the liver (upper right), chronic injury disrupts endocrine and metabolic pathways essential for bone homeostasis by reducing IGF-1 production and vitamin D activation and increasing IGFBP-1 secretion. In bone (lower panel), these gut- and liver-derived alterations induce the expression of osteoclast-related genes by activating the ROS-TFEB and IGFBP-1/ITGB1 signalling pathways. CLD, chronic liver disease; FGF19, fibroblast growth factor 19; FXR, farnesoid X receptor; IGF-1, insulin-like growth factor 1; IGFBP-1, insulin-like growth factor-binding protein 1; ITGB1, integrin β1; LPS, lipopolysaccharide; OPG, osteoprotegerin; RANKL, receptor activator of NF-κB ligand; ROS, reactive oxygen species; TFEB, transcription factor EB.

### Bone destruction is mediated by inflammation and oxidative stress

At the signalling level, inflammatory cytokines and oxidative stress further exacerbate this imbalance. TNF-α is a central inflammatory driver. It promotes osteoclast precursor maturation and activates NF-κB, thereby suppressing osteogenic differentiation and mineralisation.^65^ It also enhances osteoclastogenic signalling through the MAPK pathway, reinforcing bone resorption. IL-1β and IL-6 upregulate osteoclast-related gene expression through pathways such as NF-κB/STAT, synergistically accelerating bone loss. Recent comparative research using human-derived osteogenic models has demonstrated that TNF-α, IL-1β, and IL-6 all inhibit osteoblast mineralisation. Among them, IL-1β and TNF-α exhibit the strongest inhibitory effects, accompanied by decreased alkaline phosphatase activity. However, these cytokines exhibit limited effects on cell proliferation and the basal expression of some osteogenic marker genes.^66^

ROS also play a dual role in bone metabolism. Physiological levels of ROS are indispensable for mediating vital signalling pathways in fracture healing.^67^ Excessive ROS can directly damage osteoblast mitochondria and induce apoptosis,^68^ while also activating signalling pathways such as NF-κB and ERK/MAPK to stimulate osteoclast differentiation.^69^ ROS further promote abnormal collagen cross-linking, reducing bone mechanical strength and increasing fracture risk.^70^ Notably, a positive feedback loop exists between inflammation and ROS.^71^ Pro-inflammatory factors amplify ROS production, which in turn enhances inflammatory signalling, synergistically driving the persistent progression of bone metabolism imbalance.^72^

### Hormonal disruptions

#### Abnormal vitamin D metabolism

Vitamin D is essential for calcium-phosphorus homeostasis, and the liver plays a pivotal role in its metabolism by converting vitamin D to 25-hydroxyvitamin D [25(OH)D]. In CLD, hepatocyte injury directly suppresses 25-hydroxylase activity and impairs the synthesis of vitamin D-binding protein, compromising the systemic transport of 25(OH)D,^73^ ultimately leading to a significant decline in intestinal calcium absorption efficiency. Studies have shown that approximately one-third of patients with CHB have vitamin D insufficiency, with nearly half exhibiting deficiency.^74^ Moreover, as cirrhosis progresses, 25(OH)D levels decline further, particularly in patients with Child-Pugh class C cirrhosis.^75^

Long-term cholestasis in patients with CLD can lead to a decrease in intestinal bile acid concentrations, hindering the absorption of fat-soluble vitamins, thereby further exacerbating vitamin D deficiency. It is noteworthy that vitamin D deficiency itself can lead to secondary hyperparathyroidism, which promotes osteoclast activity and inhibits osteoblast function, creating a vicious cycle.^76^ Chronic inflammation can disrupt the secretion patterns of parathyroid hormone and calcitonin,^77^ affecting calcium-phosphorus homeostasis and bone remodelling, thereby further impairing bonemetabolism.

#### Sex hormone metabolism disorder

Sex hormones directly regulate bone metabolism through actions on bone cells and immune modulation. Oestrogen, by binding to its receptors, inhibits RANKL, upregulates OPG, and reduces the expression of sclerostin,^78^ promoting osteoblast differentiation and reducing osteoclast formation. It can also induce osteoclast apoptosis, thus decreasing bone resorption.^79^ Testosterone, on the other hand, promotes trabecular bone formation through androgen receptor signalling in osteoblasts and osteocytes.^80^

CLD causes persistent hepatocyte dysfunction and can disrupt the hypothalamic-pituitary-gonadal axis, leading to reduced sex hormone secretion and abnormal synthesis of sex hormone-binding globulin. Oestrogen deficiency can also interfere with lipid metabolism, promoting the development of osteoporosis.^81^ It is important to note that low oestrogen or low testosterone levels, in conjunction with ROS, can create a synergistic amplifying effect. Oestrogen deficiency can upregulate pro-inflammatory factors such as IL-1, IL-6, and TNF-α,^82^ further exacerbating the imbalance in bone metabolism. Fig. 3 illustrates the mechanistic links between inflammation, oxidative stress, and endocrine disturbances. In addition, individual liver diseases have distinct pathogenic mechanisms (Table 1).

**Fig. 3:**
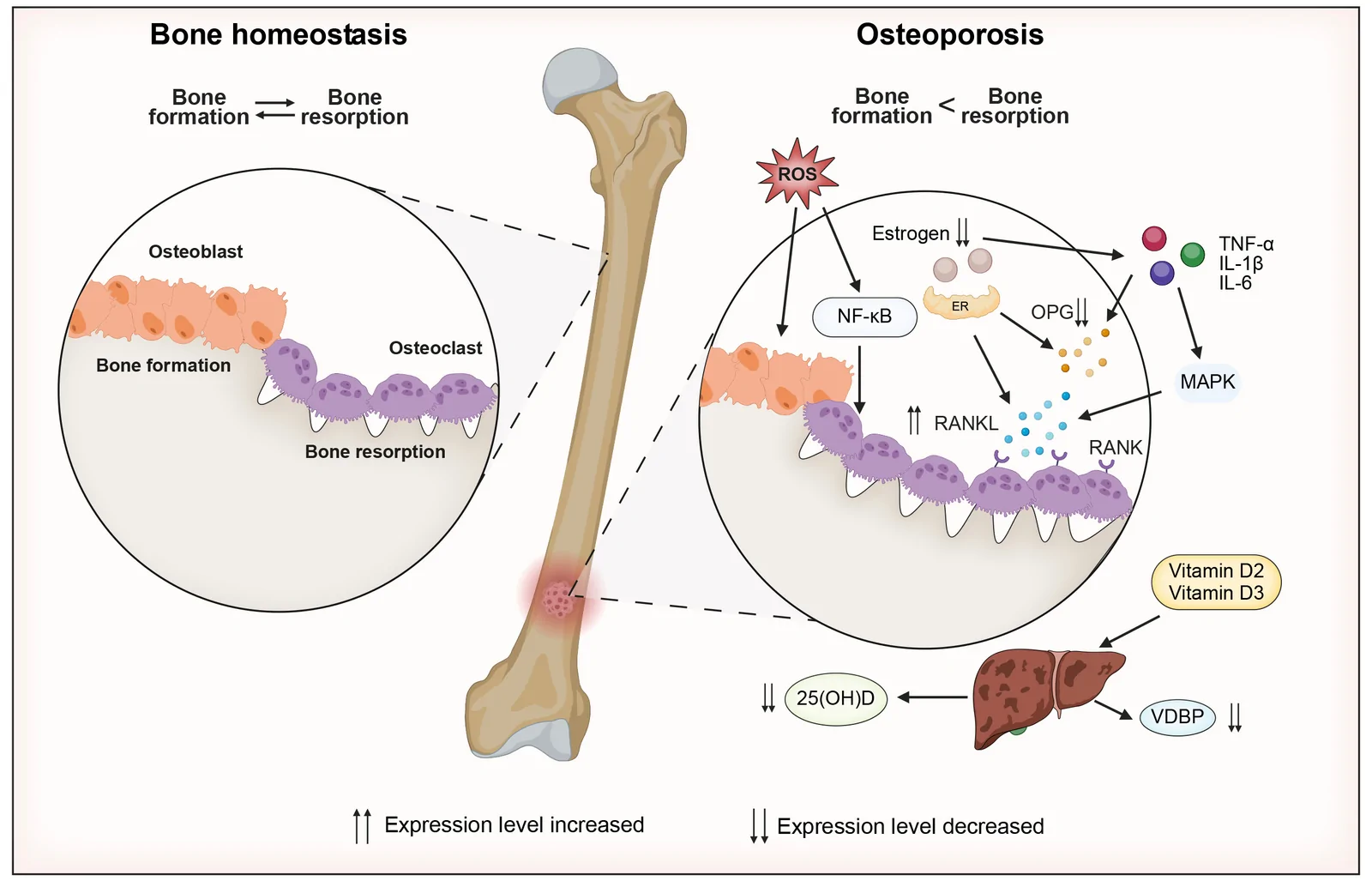
Diagram of "inflammation-oxidative stress-endocrine disorder" triple pathway. This figure summarises the mechanisms by which inflammation, oxidative stress, and endocrine disturbances contribute to liver-related osteoporosis. Pro-inflammatory cytokines and excessive ROS promote osteoclastogenesis and impair osteoblast function through NF-κB, MAPK-, and RANKL/OPG-related pathways. Meanwhile, oestrogen deficiency and impaired hepatic vitamin D metabolism further exacerbate bone loss. Together, these abnormalities shift bone remodelling toward excessive resorption and osteoporosis. 25(OH)D, 25-hydroxyvitamin D; ER, oestrogen receptor; OPG, osteoprotegerin; RANK, receptor activator of NF-κB; RANKL, receptor activator of NF-κB ligand; ROS, reactive oxygen species; VD2, vitamin D2; VD3, vitamin D3; VDBP, vitamin D-binding protein.

**Table 1:** Aetiology-specific mechanisms contributing to osteoporosis in chronic liver diseases.

Disease	Major driver	Core mechanism	Key consequences
**CHB**			
	Antiviral therapy (TDF/ADV)	Proximal tubular dysfunction → phosphate wasting.^32^ Impaired vitamin D activation and calcium-sensing signalling → secondary hyperparathyroidism.^108^ Signalling pathways involving cell migration, G-protein coupled receptors, and Toll-like receptors.^109^	Hypophosphatemia; increased bone turnover; BMD loss
	Chronic immune activation	Persistent antigen stimulation → TNF-α/IL-1β/IL-6 elevation.^110^ Treg-Th17 imbalance,^111^ with IL-17-RANKL coupling.^112^	Osteoclastogenesis Bone resorption predominance
**MASLD**			
	Insulin resistance	Defective insulin signalling + NF-κB/JNK activation sustains low-grade inflammation.^113^^,^^114^ Adipose dysfunction with macrophage infiltration.^113^	Increased resorption, impaired formation. Higher fragility risk
	Adipokine dysregulation	Leptin resistance blunts bone-anabolic signalling.^115^ Chemerin excess suppresses Wnt/β-catenin/Notch and favours osteoclast differentiation.^116^	Reduced osteoblast activity. Increased osteoclast activity.
	Lipotoxicity/mitochondrial dysfunction	Excess FFAs → mitochondrial stress/β-oxidation failure → lipid accumulation + mitochondrial dysfunction.^117^^,^^118^ ROS production.^119^	Osteoblast impairment. Osteoclast activation. Accelerated bone loss.
**ALD**			
	Alcohol-driven remodelling shift	Alcohol increases RANKL and M-CSF and disrupts RANKL–RANK–OPG via immune activation[120], [121], [122]	Enhanced osteoclast activity. Net bone resorption.
	Direct anti-osteoblast effects	Ethanol/acetaldehyde suppress osteoblast proliferation/differentiation and matrix protein synthesis.^123^	Reduced bone formation. Bone loss.
	Malabsorption & fall risk	Increased gut permeability → impaired fat-soluble vitamin/phosphate absorption.^121^ CNS/cerebellar dysfunction → falls.^124^	Impaired mineralization. Fracture risk via falls
**AIH**			
	Immune dysregulation	Pro-inflammatory cytokines (↑TNF-α/IL-1β/IL-6) promote RANKL-dependent osteoclast activation	Resorption-dominant remodelling. Net bone loss.
	Corticosteroid side effects	Negative calcium balance (inhibited intestinal calcium absorption and increased urinary calcium excretion). Direct bone effects (suppressed osteoblast activity with enhanced osteoclast activity)^125^	Accelerated BMD loss. Increased fragility fracture risk.

ADV, adefovir dipivoxil; AIH, autoimmune hepatitis; ALD, alcohol-associated liver disease; BMD, bone mineral density; CHB, chronic hepatitis B; CNS, central nervous system; FFA, free fatty acid; MASLD, metabolic dysfunction-associated steatotic liver disease; M-CSF, macrophage colony-stimulating factor; OPG, osteoprotegerin; RANK, receptor activator of NF-κB; RANKL, receptor activator of NF-κB ligand; ROS, reactive oxygen species; TDF, tenofovir disoproxil fumarate.

## Prevention and early detection of liver disease-related osteoporosis

### Screening and diagnosis

Osteoporosis often develops insidiously and lacks specific symptoms, so it is frequently identified only after fragility fractures occur. Therefore, establishing an early risk identification system for high-risk populations – particularly the elderly, postmenopausal women, men with hypogonadism, patients with cirrhosis, patients with AIH receiving prolonged glucocorticoid therapy, and those receiving long-term TDF treatment – is crucial. Currently, dual-energy X-ray absorptiometry (DXA) remains the first-line tool for assessing BMD in patients with CLD.[83], [84], [85] However, BMD alone may underestimate fracture risk and is less sensitive to early microstructural changes. Trabecular bone score complements DXA by evaluating bone microarchitecture and identifying early fragility.^86^^,^^87^ Recent evidence further suggests that muscle strength–related parameters should be incorporated into the skeletal risk assessment framework for patients with steatotic liver disease. Yokoyama *et al.* reported that reduced grip strength was significantly associated with lower BMD and proposed that patients with MetALD and impaired grip strength should be prioritised for BMD assessment.^30^ Therefore, incorporating bone quality indicators (such as bone microstructure and mineralisation) and functional factors (such as muscle strength) into a comprehensive assessment may more accurately reflect the bone fragility status of patients with CLD.^88^^,^^89^ High-resolution peripheral quantitative CT enables precise assessment of fracture risk by constructing three-dimensional images of bones and accurately measuring bone quality parameters such as cortical thickness and trabecular microstructure.^90^ In patients receiving long-term antiviral therapy, serum 25(OH)D may be incorporated into longitudinal monitoring as an indicator of hepatic-bone metabolic status. For perimenopausal women and men with low testosterone, a stratified evaluation approach should be implemented in conjunction with sex hormone profile analysis.

Classical bone turnover markers (BTMs) such as serum PINP (procollagen type I N-terminal propeptide) and plasma β-CTX (β-C-terminal telopeptide of type I collagen) can detect early changes in bone formation and resorption, aiding treatment monitoring and compliance assessment.^91^ Emerging biomarkers, including microRNAs (miR-23b-3p,^92^ miR-197-3p^93^) and proteomic signatures (*e.g,* sclerostin, adiponectin, creatine kinase B-type),^94^ show potential but remain investigational and are not yet suitable for routine practice. Therefore, the risk assessment of liver disease-related osteoporosis should integrate multidimensional information including imaging, metabolic, and molecular data to establish a more precise dynamic monitoring pathway.

### Prevention strategies

Prevention of liver disease-related osteoporosis should rely on comprehensive measures including nutritional optimisation, lifestyle interventions, and management of the underlying liver disease. Emphasis should be placed on a balanced diet and adequate protein intake, while ensuring sufficient calcium and vitamin D intake.^83^ For patients with 25(OH)D levels <20 ng/ml, vitamin D supplementation should be initiated. A prospective study showed that, in adherent patients with cirrhosis and vitamin D deficiency, a baseline 25(OH)D-guided vitamin D3 regimen (2,000 IU/day for 25(OH)D levels >15 to ≤25 ng/ml and 4,000 IU/day for levels ≤15 ng/ml), with reassessment every 3 months, safely corrected deficiency. For individuals with severe liver impairment, conventional vitamin D supplementation shows limited efficacy. Calcifediol may be preferred to compensate for deficient hepatic hydroxylation activity and rapidly increase serum 25(OH)D levels.^95^

A healthy lifestyle also plays a significant role in reducing fracture risk. Smoking cessation should be encouraged in all patients, and complete alcohol abstinence is essential in ALD. While regular weight-bearing and resistance exercise may benefit clinically stable patients, those with decompensated cirrhosis – especially with ascites, hepatic encephalopathy, or recent variceal bleeding – should preferentially engage in low-intensity aerobic and supervised functional training, with high-intensity weight-bearing exercise avoided to minimise fall, trauma, and bleeding risk. These measures aim to enhance bone strength through mechanical stimulation and improve muscle strength to reduce fall risk. For patients with CLD, optimising the treatment of underlying conditions through standardised antiviral and immunomodulatory therapies can effectively control chronic inflammation and metabolic disorders, thereby mitigating negative impacts on bone metabolism at the source.^3^ Therefore, establishing a multidimensional management system incorporating primary disease control, nutritional support, and scientifically guided lifestyle interventions can effectively reduce the risk of fractures in patients with CLD (Fig. 4).

**Fig. 4:**
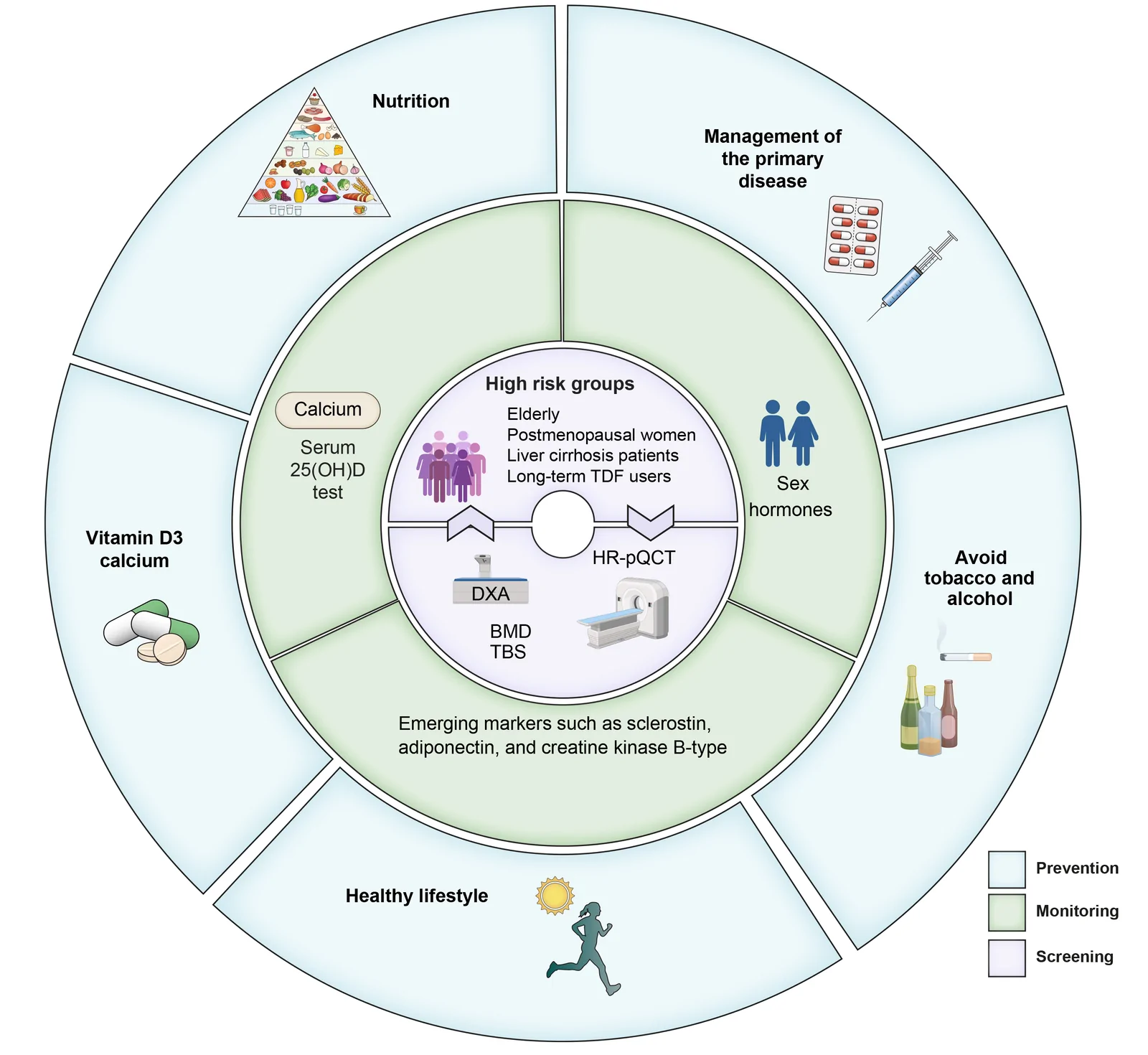
Flowchart of screening-monitoring-prevention. This figure outlines a comprehensive framework for managing liver-related osteoporosis. Screening: Identifies high-risk groups (*e.g*. those with cirrhosis, postmenopausal women, TDF users) using advanced bone assessments like DXA, TBS, and HR-pQCT. Monitoring: Evaluates bone health via serum 25(OH)D, sex hormones, and novel biomarkers (*e.g*. sclerostin, adiponectin). Prevention: Emphasises adequate nutrition, calcium/vitamin D3 supplementation, healthy lifestyle, and treating the primary liver disease. Collectively, this integrated strategy enables early detection, dynamic assessment, and prevention of bone loss in chronic liver disease. 25(OH)D, 25-hydroxyvitamin D; BMD, bone mineral density; DXA, dual-energy X-ray absorptiometry; HR-pQCT, high-resolution peripheral quantitative computed tomography; TBS, trabecular bone score; TDF, tenofovir disoproxil fumarate.

## Treatment of liver disease-related osteoporosis

### Bone resorption inhibitors and bone formation promoters

Treatment of osteoporosis in patients with CLD requires a balance between anti-osteoporotic efficacy and preservation of liver function. Bisphosphonates are considered effective bone resorption inhibitors for treating patients with CHB and those with cirrhosis. They are not metabolised by the liver and are primarily cleared by the kidneys, thus posing minimal direct toxicity to the liver when kidney function is normal. Evidence in Child–Pugh C cirrhosis or severely decompensated cirrhosis remains limited, and treatment should therefore be used cautiously with close monitoring. Additionally, denosumab, a fully human monoclonal antibody targeting RANKL, is not metabolised by the liver or kidneys and is safe to use in patients with CLD. The DELTA study demonstrated that denosumab was non-inferior to zoledronic acid in improving BMD, alongside a markedly lower incidence of adverse events (14.3%). Furthermore, denosumab favourably altered the ratio of serum alkaline phosphatase to the upper limit of normal, suggesting additional regulatory benefits on biochemical markers of bone metabolism.^96^ Studies have shown that in patients with CHB receiving TDF treatment for more than a year, denosumab significantly improves lumbar spine and hip BMD compared to bisphosphonates, along with a substantial decrease in bone resorption markers.^97^ Denosumab may be considered in selected patients with TDF-associated bone loss, particularly when renal function, adherence, or bisphosphonate tolerance is a concern; however, longer-term safety and fracture-outcome data in CLD populations remain limited.

In contrast, selective oestrogen receptor modulators (such as tamoxifen) and bone formation promoters (such as teriparatide) have more limited roles. Although tamoxifen may improve BMD in postmenopausal women, its potential hepatotoxicity warrants caution. Clinical observations suggest that long-term tamoxifen use is associated with an increased risk of liver function abnormalities, with approximately 40% of long-term users developing radiological evidence of fatty liver.^43^^,^[98], [99], [100] Therefore, tamoxifen is not recommended as a first-line therapy and should be considered only in selected perimenopausal women with contraindications to bisphosphonates and denosumab, with close monitoring of liver function during treatment. While teriparatide outperforms bisphosphonates in increasing bone mass and reducing fracture risk,^101^ its primary clearance depends on the liver. There is insufficient evidence regarding its safety in patients with impaired liver function, necessitating careful evaluation. Notably, hormone replacement therapy (HRT) can serve as an alternative option for patients with impaired liver function. Although oral oestrogen may exacerbate the hepatic metabolic burden, transdermal administration routes (*e.g*. patches or transdermal gels) effectively circumvent this metabolic process.^102^ Consequently, for patients with mild or well-compensated CLD, transdermal HRT is considered a relatively safe and effective strategy for maintaining BMD and preventing fractures, provided that hepatic biochemical parameters are closely monitored.

### Novel osteoporosis medications

Shifting toward more precise treatment modalities, recent studies have proposed innovative targeted drug delivery strategies, all of which are currently at the preclinical stage. Regarding general novel therapies designed to address the inflammatory and cellular characteristics of the osteoporotic bone microenvironment, multi-target precision interventions have demonstrated significant advantages. One approach utilises alendronate-modified liposomes to co-deliver arginine and metformin directly to the bone microenvironment, enabling precise local accumulation and modulation of osteogenesis and osteoclastogenesis. For instance, the calcium-magnesium-supplemented nano-platform, a bone-targeted nanocarrier functionalised with alendronate ligands, enables precise drug delivery to bone tissue, alleviates inflammatory stress by inhibiting the NF-κB signalling pathway, and promotes osteogenic differentiation.^103^ Furthermore, bioinspired and intelligent responsive technologies, which are designed to mimic biological systems and respond dynamically to specific microenvironmental cues, provide a more precise strategy for cross-organ regulation. Engineered extracellular vesicles derived from mesenchymal stem cells achieve bidirectional regulation of bone metabolism by delivering RANK molecules to neutralise excessive RANKL, while releasing miRNA-21a-5p to inhibit osteoclast differentiation and promote osteogenesis.^104^ Cui *et al.* developed a bone-targeting bioinspired nanogel that further enhances this approach. It utilises CXCR4-mediated targeting to accumulate in the bone microenvironment and intelligently releases synthetic parathyroid hormone, which promotes bone formation, in response to the acidic microenvironment generated by osteoclasts, thereby achieving multi-target dynamic equilibrium remodelling.^105^

Highly specific interventions targeting liver disease-related osteoporosis have also made preclinical breakthroughs. An innovative targeted approach utilises alendronate-modified liposomes to co-deliver arginine and metformin directly to the bone. Interestingly, the inevitable hepatic accumulation of these liposomes stimulates the hepatic secretion of lecithin-cholesterol acyltransferase, optimising reverse cholesterol transport to indirectly enhance bone homeostasis from metabolic origins.^106^ Additionally, the novel tetrahydro-β-carboline derivative Y2641, a dual inhibitor of RANKL/TNF-α, not only reduces inflammation and bone resorption but also remodels subchondral bone structure, providing a new therapeutic approach for antiviral drug-associated bone damage.^107^

These findings suggest that the treatment of liver disease–related osteoporosis is shifting toward precision drug delivery and cross-organ metabolic regulation. However, the clinical translation of these preclinical strategies remains uncertain. Although they may theoretically offer advantages over existing therapies, including enhanced bone targeting, reduced systemic exposure, and coordinated modulation of inflammation, osteoclastogenesis, and osteogenesis, there is currently no evidence that they provide more durable long-term clinical benefits than established treatments. In addition, challenges related to large-scale manufacturing, formulation stability, and safety may limit their clinical applicability, particularly in patients with CLD. Future research should therefore focus on direct comparisons with existing therapies and rigorous safety evaluation to facilitate clinical translation (Fig. 5).

**Fig. 5:**
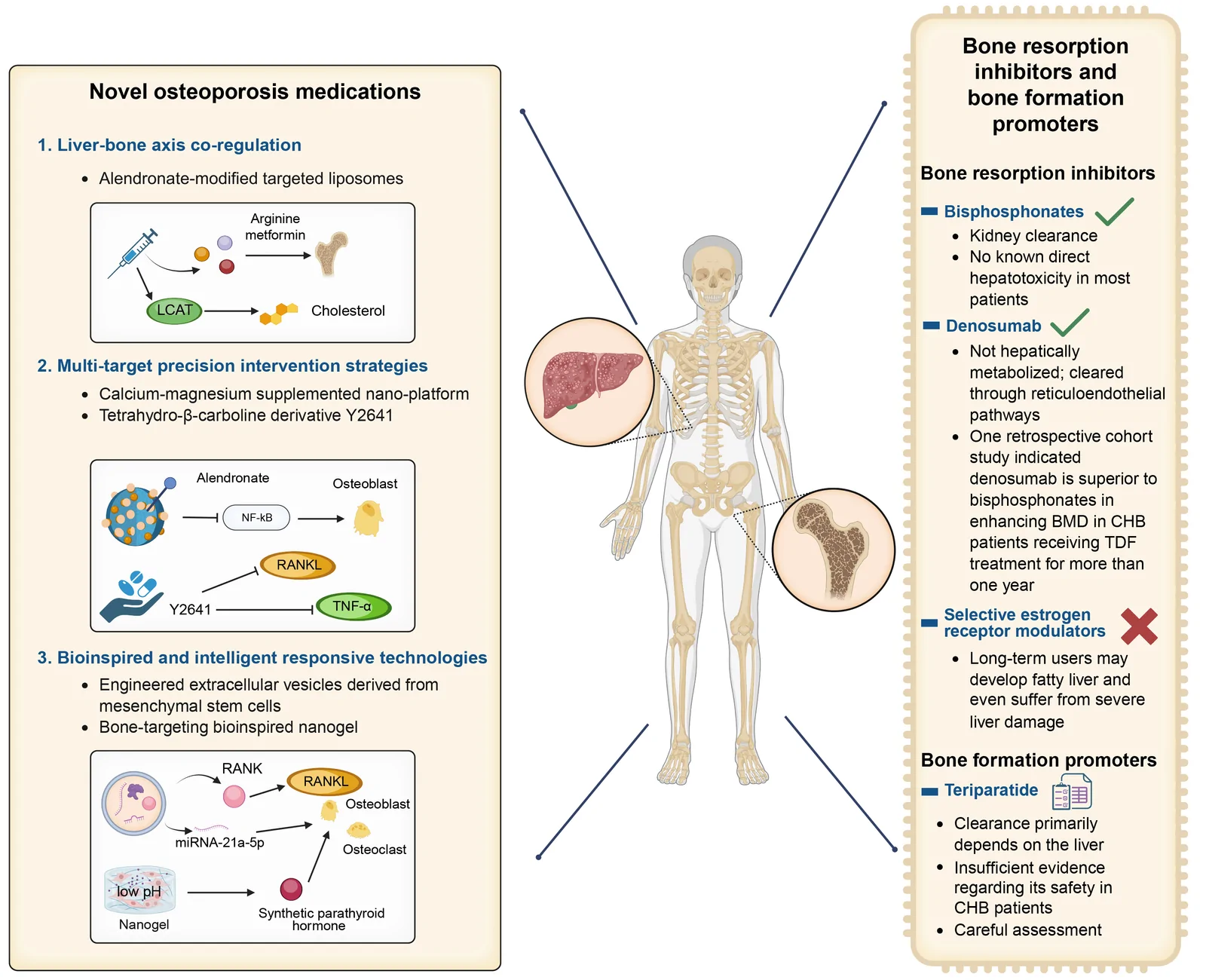
Clinical treatment and novel therapies for osteoporosis in patients with CLD. This figure summarises current and emerging therapeutic strategies for CLD-related osteoporosis. The right panel outlines currently available agents, including antiresorptive therapies (bisphosphonates and denosumab) and an anabolic agent (teriparatide), whereas selective oestrogen receptor modulators may be limited by potential hepatic safety concerns. The left panel highlights emerging approaches, including liver–bone axis co-regulation, multi-target precision interventions, and bioinspired intelligent responsive technologies. Representative strategies include alendronate-modified targeted liposomes, calcium–magnesium-supplemented nanoplatforms, Y2641, engineered extracellular vesicles, and bone-targeting nanogels. Collectively, these therapies reflect a shift from conventional anti-osteoporotic treatment toward mechanism-based and targeted interventions for CLD-related bone loss. BMD, bone mineral density; CHB, chronic hepatitis B; CLD, chronic liver disease; LCAT, lecithin-cholesterol acyltransferase; miRNA, microRNA; RANK, receptor activator of NF-κB; RANKL, receptor activator of NF-κB ligand; TDF, tenofovir disoproxil fumarate.

### Differences in osteoporosis medications for different liver diseases

There are significant differences in the selection and utilisation patterns of osteoporosis medications among patients with CLD of varying aetiologies, which are closely related to drug-disease interactions and disease pathogenesis (Table 2).

**Table 2:** Differences in osteoporosis medication among patients with different liver diseases aetiologies.

Liver disease type	Core treatment principles	Therapeutic regimen	Contraindicated/use with caution	Adjunctive management
**CHB**	Optimise antiviral therapy and minimise drug-related bone loss.	TAF is preferred over TDF in patients at high fracture risk. Bisphosphonates or denosumab may be selected according to renal function.	Avoid unnecessary polypharmacy to reduce hepatic burden.	Calcium/vitamin D supplementation. Fracture risk screening. DXA/BTM monitoring.
**MASLD**	Integrate osteoporosis treatment with metabolic management.	Bisphosphonates or denosumab according to standard osteoporosis indications. Optimise metabolic therapy. Metformin,[126], [127], [128] GLP-1 receptor agonists,^129^ and selected SGLT2 inhibitors^130^^,^^131^ may provide additional skeletal benefit in appropriate patients. Emerging evidence suggests that GLP-1 receptor agonists may confer potential bone-protective effects, including a possible reduction in fracture risk; however, the overall certainty of the evidence remains limited.^132^	Thiazolidinediones (*e.g*. pioglitazone) should be used cautiously because of increased fracture risk.^133^ Observational data have associated tirzepatide with a higher recorded risk of osteoporosis/fracture outcomes than some other GLP-1 receptor agonists; this finding requires confirmation and should not be interpreted as definitive causality.^134^	Weight reduction. Exercise. Glycaemic/lipid control. Vitamin D/calcium supplementation.
**ALD**	Correct nutritional deficiency and enforce alcohol abstinence.	Due to poor adherence to oral medications, intravenous bisphosphonates or denosumab injections are more feasible.^135^	Ongoing alcohol exposure, malnutrition, and poor adherence may limit efficacy and safety. Use oral agents cautiously in unstable patients.	Complete alcohol abstinence. Nutritional rehabilitation. Vitamin D/calcium supplementation. Fall prevention.
**AIH**	Minimise glucocorticoid exposure while maintaining disease control	Bisphosphonates remain first-line for glucocorticoid-induced osteoporosis.^136^ Teriparatide and denosumab are superior to oral bisphosphonates in reducing vertebral fractures in patients with corticosteroid-induced osteoporosis.^136^	Long-term/high-dose corticosteroids markedly increase fracture risk. Treatment choice should consider immunosuppressive regimen and liver status.	Steroid-sparing strategies. Vitamin D/calcium supplementation. Regular BMD/BTM monitoring.

AIH, autoimmune hepatitis; ALD, alcohol-associated liver disease; BMD, bone mineral density; BTM, bone turnover marker; CHB, chronic hepatitis B; DXA, dual-energy X-ray absorptiometry; GLP-1, glucagon-like peptide-1; MASLD, metabolic dysfunction-associated steatotic liver disease; SGLT2, sodium-glucose cotransporter 2.

## Conclusion

CLD is closely associated with osteoporosis. Its pathogenesis is not driven by a single pathway, but by an imbalance in bone remodelling mediated through the interplay of inflammation, endocrine dysfunction, and the liver-bone and liver-gut-bone axes. As liver disease progresses, the risk of osteoporosis gradually increases. Although the link between CLD and osteoporosis is increasingly recognised, further studies are needed to define aetiology-specific molecular mechanisms and to evaluate the efficacy and safety of emerging anti-osteoporotic therapies across different degrees of hepatic dysfunction. Early screening and intervention for osteoporosis are critical components in the management of patients with liver disease. Current screening approaches should comprehensively integrate BMD, bone quality, muscle strength, and emerging biomarkers to enable multidimensional assessment of osteoporosis risk. Moreover, novel therapeutic strategies are gradually being developed, with targeted drug delivery and cross-organ metabolic regulation emerging as key research priorities for the future. To optimise the management of liver disease-associated osteoporosis, future research should prioritise multicentre, prospective studies and the development of personalised treatment strategies tailored to different liver diseases, with the aim of reducing bone loss and fracture risk and improving patients' long-term prognosis and quality of life.

## Abbreviations

25(OH)D, 25-hydroxyvitamin D; 25(OH)D3, 25-hydroxyvitamin D3; AIH, autoimmune hepatitis; ALD, alcohol-associated liver disease; BMD, bone mineral density; BTM/BTMs, bone turnover marker(s); CHB, chronic hepatitis B; CLD, chronic liver disease; DXA, dual-energy X-ray absorptiometry; HRT, hormone replacement therapy; IGF-1, insulin-like growth factor 1; IGFBP-1/IGFBP1, insulin-like growth factor-binding protein 1; MASLD, metabolic dysfunction-associated steatotic liver disease; miRNA/miRNAs, microRNA(s); OPG, osteoprotegerin; RANK, receptor activator of NF-κB; RANKL, receptor activator of NF-κB ligand; ROS, reactive oxygen species; TDF, tenofovir disoproxil fumarate.

## Authors’ contributions

WJW: Study concept and design, Visualization, and Writing - Original Draft. GYL: Conceptualization, Writing - Original Draft. BY, TQY, HLS, PP, and YW: Writing – review & editing. PP and YW: Study supervision. All authors provided critical revisions and approved the final version of the manuscript.

## Financial support

This work was supported by the National Science and Technology Major Project on the Prevention and Control of Emerging, Acute, and Major Infectious Diseases (Grant Number: 2025ZD01905903) and Intramural program of the National Institutes of Health, USA.

## Conflict of interest

Please refer to the accompanying ICMJE disclosure forms for further details.
